# The radiological interpretation of possible microbleeds after moderate or severe traumatic brain injury: a longitudinal study

**DOI:** 10.1007/s00234-021-02839-z

**Published:** 2021-10-31

**Authors:** Anke W. van der Eerden, Thomas L. A. van den Heuvel, Marnix C. Maas, Priya Vart, Pieter E. Vos, Bram Platel, Bozena M. Góraj, Rashindra Manniesing

**Affiliations:** 1 Department of Radiology and Nuclear Medicine, Radboud University Medical Center, Nijmegen, Netherlands; 2 Department of Radiology & Nuclear Medicine, NE-515, Erasmus Medical Center, P.O. Box 2040, 3000 CA Rotterdam, Netherlands; 3 Department of Epidemiology and Biostatistics, Radboud University Nijmegen, Nijmegen, Netherlands; 4 Department of Neurology, Santiz-Slingeland Hospital, Doetinchem, Netherlands

**Keywords:** Brain injuries, Traumatic, Cerebral hemorrhage, Traumatic, Diffuse axonal injury,, Magnetic resonance imaging, Longitudinal studies

## Abstract

**Introduction:**

In order to augment the certainty of the radiological interpretation of “possible microbleeds” after traumatic brain injury (TBI), we assessed their longitudinal evolution on 3-T SWI in patients with moderate/severe TBI.

**Methods:**

Standardized 3-T SWI and T1-weighted imaging were obtained 3 and 26 weeks after TBI in 31 patients. Their microbleeds were computer-aided detected and classified by a neuroradiologist as no, possible, or definite at baseline and follow-up, separately (*single-scan* evaluation). Thereafter, the classifications were re-evaluated after comparison between the time-points (*post-comparison* evaluation). We selected the possible microbleeds at baseline at *single-scan* evaluation and recorded their *post-comparison* classification at follow-up.

**Results:**

Of the 1038 microbleeds at baseline, 173 were possible microbleeds. Of these, 53.8% corresponded to no microbleed at follow-up. At follow-up, 30.6% were possible and 15.6% were definite. Of the 120 differences between baseline and follow-up, 10% showed evidence of a pathophysiological change over time. Proximity to extra-axial injury and proximity to definite microbleeds were independently predictive of becoming a definite microbleed at follow-up. The reclassification level differed between anatomical locations.

**Conclusions:**

Our findings support disregarding possible microbleeds in the absence of clinical consequences. In selected cases, however, a follow-up SWI-scan could be considered to exclude evolution into a definite microbleed.

**Supplementary Information:**

The online version contains supplementary material available at 10.1007/s00234-021-02839-z.

## Introduction

The yearly incidence of traumatic brain injury (TBI) is around 300 per 100,000 persons [1, 2]. One of the injury types encountered in these patients is cerebral microbleeds (CMBs) [3–7]. Traumatic CMBs represent accumulations of hemosiderin-containing macrophages, usually caused by traumatic vascular injury [3]. They are associated with traumatic axonal injury [8, 9]. Depending on their distribution, both are negatively associated with prognosis [3, 10–12].

The Microbleed Anatomical Rating Scale (MARS) [13] classifies microbleeds as “definite” or “possible” microbleeds within pre-defined anatomical brain-regions, with a high interobserver agreement for the assessment of definite microbleeds [4, 13, 14]. The assessment of possible microbleeds with GRE T2*-weighted 1.5-T MRI demonstrates substantial observer variability, due to equivocality in their interpretation as a CMB versus an alternative source of focal low signal intensity [13, 14]. Therefore, to improve the reliability of microbleed rating, it is suggested to regard these possible microbleeds as “no finding” in a research setting [13]. In radiological practice, however, these equivocal findings are encountered daily, and the decision on how to interpret them in an individual patient is less straightforward. Indeed, the authors of the Brain Observer MicroBleed Scale paper suggested the performance of a longitudinal study to determine if possible microbleeds mature into definite CMBS (named “uncertain” and “certain” in their paper) [14].

In an attempt to augment the certainty of the radiological interpretation of possible microbleeds in daily practice, the purpose of this study was to assess the longitudinal evolution of possible microbleeds on SWI in patients with moderate or severe TBI. We also evaluated possible predictors of this evolution and assessed the causes of differences.

## Methods

The data were obtained within a prospective observational long-term follow-up cohort study in consecutive patients with moderate or severe TBI (defined as head trauma resulting in Glasgow Come Scale score (GCS) 9–12 and 3–8 at the injury site, respectively), approved by Radboud University Medical Center’s Institutional Review Board. The study was performed in accordance with the ethical standards as laid down in the 1964 Declaration of Helsinki and its later amendments. All patients or their next of kin gave written informed consent. This is the second publication on this study population [9].

### Study design

**Fig. 1:**
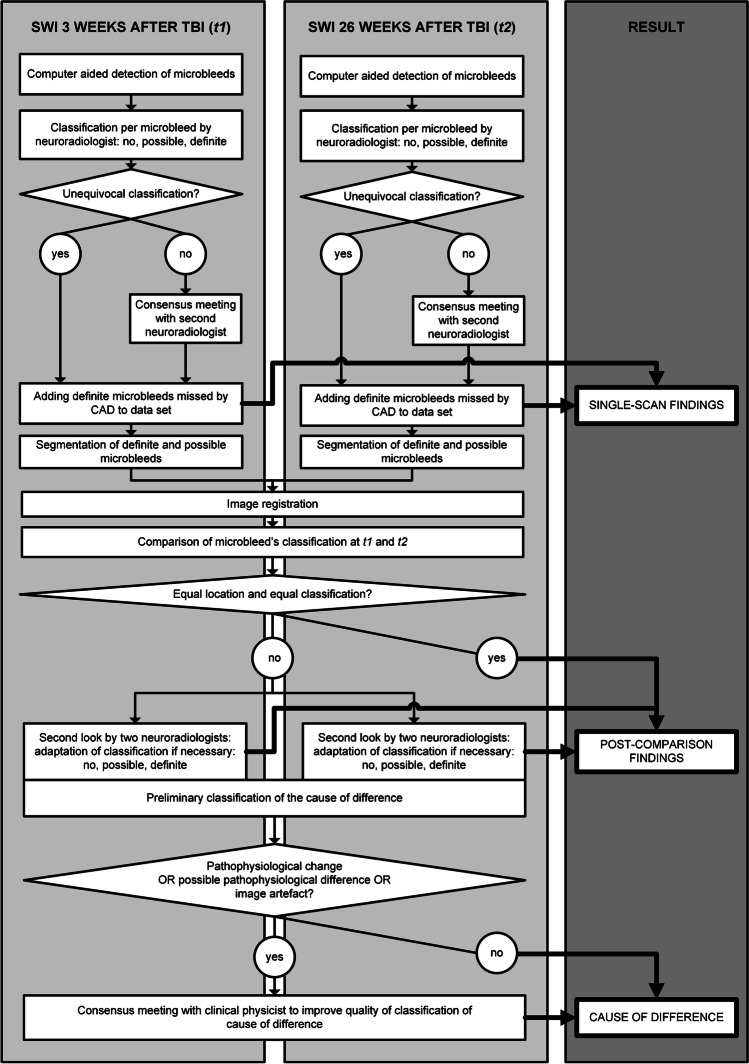
Overview of study procedure. *TBI* traumatic brain injury, *t1* 3 (2–5) weeks after TBI, t2 26 (25–28) weeks after TBI, *CAD* computer-aided detection

Figure 1 summarizes the study design comparing SWI-findings obtained median 3 (2–5 (*t1*)) and 26 (25–28 (*t2*)) weeks after TBI. It defines the terms *single-scan findings*, *post-comparison findings*, and *cause of difference* used throughout the paper. We assessed how frequently possible microbleeds detected at *t1* in the *single-scan* stage, were classified as no, possible, and definite microbleed at *t2* in the *post-comparison* stage. The *single-scan findings* at *t1* approach daily radiological practice: in a radiological setting, the decision on the interpretation of a possible microbleed needs to be made without any information on the findings at follow-up. Lacking correlation with pathological specimens, the *post-comparison findings* give information on the pathophysiological evolution: in the *post-comparison* stage the effects of interpretational uncertainty are minimized.

### Definitions

We defined definite microbleeds as small, well-defined areas of SWI-signal void at least half surrounded by brain parenchyma, with clear margins ranging from 2 to 10 mm in size in at least one plane, with distinct appearance from potential mimics (e.g., calcium and iron deposits, bone, or vessel flow voids), which were not obviously part of an intraparenchymal hemorrhage or contusion. Possible microbleeds were less well defined or less hypointense (Table 1). Due to the lack of consensus on the definition of a traumatic CMB, this definition is an adaptation from non-traumatic literature (the STRIVE criteria [15], the Greenberg recommendations [5], and the MARS guidelines [13]), ignoring their criterion of sphericalness or ovalness, as traumatic CMBs can notably be elongated in shape [6, 16], and evaluating SWI instead of GRE T2*-weighted images. We use the term “microbleed” in a methodological sense. It comprises any finding classified as possible or definite microbleed at *t1* and/or at *t2*. The term “CMB” is reserved for cerebral microbleed in a pathophysiological sense. The term “extra-axial injury score” is defined in the *Online Resource*.

**Table 1:** Definition of possible and definite microbleed

Definite microbleed	Possible microbleed
Area of SWI signal void	May be less hypo-intense
At least half surrounded by brain parenchyma	At least half surrounded by brain parenchyma
Well-defined, with clear margins	May be less well-defined
2 to 10 mm in size in at least one plane	2 to 10 mm in size in at least one plane
Distinct appearance from potential mimics (e.g., calcium and iron deposits, bone, or vessel flow voids)	The differentiation from a mimic may be uncertain, but a microbleed must be the most likely diagnosis
Not part of an intraparenchymal hemorrhage or contusion	Not part of an intraparenchymal hemorrhage or contusion

To be classified as a definite microbleed, all of the criteria in the left column had to be met. Configurations that were less hypo-intense, less well-defined, and/or differentiable with less certainty from a potential mimic were classified as a possible microbleed (right column)

### Patients and image acquisition

The selection procedure described before [9] was prospectively applied to all 211 consecutive ≥ 18-year-old patients having sustained a moderate or severe TBI < 24 h before presenting at the emergency department of Radboud University Medical Center, a level I trauma center. Finally, this yielded 31 patients with SWI and T1-weighted imaging data, obtained 3 (2–5) and 26 (25–28) weeks after TBI on a single 3-T MRI scanner (Magnetom Trio, Siemens Healthineers) using the imaging parameters summarized in Table 2. The demographic and clinical patient characteristics were reported before [9], and they had a total of 865 definite and 173 possible microbleeds distributed over the patients and over the brain as summarized in Table 3.

**Table 2:** Imaging parameters

	MPRAGE	SWI
TR (ms)	2300	27
TE (ms)	2.98	20.0
TI (ms)	900	
Flip angle (degrees)	9	15
Bandwidth (Hz/pixel)	240	120
Slice thickness (mm)	1.0	1.0
Voxel-size (mm)	1.0 × 1.0 × 1.0	1.0 × 1.0 × 1.0
FOV (mm)	256	250
TA (minutes)	5:21	7:44
Dimension	3D	3D

Adapted from [9]

*TA* acquisition time

**Table 3:** Number of microbleeds and concomitant lesions in each MARS-region 3 weeks after TBI

MARS-region	Definite microbleeds	Possible microbleeds	IPH	NHC	SAH	SDH	EDH
Frontal	314.0 (6.9)	56.7 (1.0)	22 (0)	0 (0)	5 (0)	0 (0)	0 (0)
Temporal	246.8 (7.8)	55.6 (.8)	20 (0)	2 (0)	1 (0)	0 (0)	1 (0)
Parietal	75.8 (1.8)	22.3 (.0)	2 (0)	0 (0)	7 (0)	0 (0)	0 (0)
Cerebellum	19.2 (.0)	10.3 (.0)	1 (0)	0 (0)	16 (0)	0 (0)	0 (0)
Brainstem	33.6 (.2)	5.3 (.0)	1 (0)	2 (0)	0 (0)	1 (0)	0 (0)
Optic pathways	16.7 (.0)	5.1 (.0)	0 (0)	0 (0)	0 (0)	0 (0)	0 (0)
Corpus callosum	39.9 (.0)	4.9 (.0)	3 (0)	1 (0)	0 (0)	0 (0)	0 (0)
Insula	10.2 (.0)	4.6 (.0)	1 (0)	0 (0)	0 (0)	0 (0)	0 (0)
External capsule	13.1 (.0)	3.9 (.0)	0 (0)	0 (0)	-	-	-
Occipital	32.2 (.8)	3.1 (.0)	2 (0)	0 (0)	1 (0)	0 (0)	1 (0)
Internal capsule	9.4 (.0)	2.1 (.0)	1 (0)	0 (0)	-	-	-
Basal ganglia	25.7 (.0)	2.0 (.0)	2 (0)	0 (0)	-	-	-
Deep and periventricular white matter	24.9 (.2)	1.5 (.0)	1 (0)	0 (0)	-	-	-
Thalamus	4.5 (.0)	.0 (.0)	0 (0)	0 (0)	-	-	-
Whole brain	865 (24)	173 (5)	56 (1)	5 (0)	30 (1)	1 (0)	2 (0)

Values are total number of lesions (median number of lesions per patient) at *t1* at *single-scan evaluation*. Only lesions that met the criteria described in the *Online Resource* were counted; e.g., epidural hematomas of < 1-cm thickness were excluded from these numbers. No values regarding extra-axial hemorrhage are presented for the regions “deep and periventricular white matter,” “basal ganglia,” “thalamus,” “internal capsule,” and “external capsule,” as due to their location they cannot be adjacent to extra-axial injury

The anatomical MARS regions are presented in descending order of number of possible microbleeds at *t1*

*MARS* Microbleed Anatomical Rating Scale [13], *TBI* traumatic brain injury, *IPH* intraparenchymal hemorrhages, *NHC* non-hemorrhagic contusions, *SAH* subarachnoid hemorrhages, *SDH* subdural hematomas, *EDH* epidural hematomas

### Concomitant injury

Information on concomitant injury, i.e., traumatic injury other than microbleeds, was collected at *t1* as described in the *Online Resource*.

### MARS atlas

In order to localize each microbleed or concomitant injury, we manually segmented the standard brain in Montreal Neurological Institute space [17] into the regions specified in the MARS scoring template [13]. The segmentation procedure was described previously [9]. We compounded putamen, globus pallidus, and nucleus caudatus into the region “basal ganglia.”

### Microbleed evaluation

Figure 1 summarizes the microbleed evaluation process. The *Online Resource* describes further details.

#### Single-scan findings

A computer-aided detection (CAD) system, described previously [18], evaluated the scans obtained at *t1* and *t2*, separately. The CAD system was set up with a high sensitivity, such that the chances of missing any possible microbleed was very low [18]. Blinded for the other time-point, AE (neuroradiologist, 7 years of experience) classified each CAD-proposed microbleed as no, possible, or definite microbleed, deciding on CAD detections with the slightest doubt in consensus meetings with BG (neuroradiologist, 33 years of experience). Through visual screening, AE manually added definite microbleeds missed by the CAD system to the data set. Then, each definite or possible microbleed was automatically segmented using intensity-based volume-constrained region growing. Each microbleed was allocated to a MARS-region using the non-linear registration tool FNIRT [19, 20]. In the rest of the paper, when referring to *single-scan findings*, we refer to our classification at this stage.

#### Post-comparison findings

The SWI images with the segmentations at *t1* and *t2* were registered using the linear registration tool FLIRT [21, 22], and the segmentations were automatically compared between the time-points. In order to improve the quality of our microbleed evaluation, we used classification differences between the time-points to select microbleeds deserving a second look. AE and BG re-evaluated each of these microbleeds, based on the configuration per scan, not taking into account temporal pathophysiological assumptions. In case of equivocality, the original classification was maintained. In the rest of the paper, when referring to *post-comparison findings*, we refer to our classification at this stage.

#### Causes of differences

Each microbleed with a different *single-scan* classification between the time-points was reviewed by AE and BG to identify the *cause of difference*, selecting one of the options listed in Table 4, including “pathophysiological difference.” The latter was only used if, when comparing the SWI scans, it was highly likely that the microbleed pathophysiologically changed from the first to the second scan.

**Table 4:** Causes of classification differences between *t1* and *t2*

Etiological category	Subcategory	Number of microbleeds (%)	
		From possible to no (*n* = 93)	From possible to definite (*n* = 27)
Pathophysiological difference^a^		7 (8%)	5 (19%)
Possible pathophysiological difference^b^		2 (2%)	5 (19%)
Interpretation-related	Equivocal classification of microbleed^c^	4 (4%)	1 (4%)
	Missed by CAD and expert	0 (0%)	0 (0%)
	Misinterpreted	39 (42%)	5 (19%)
Technique-related	Misregistration or missegmentation	2 (2%)	7 (26%)
	Artifact hampering the interpretation^d^	36 (39%)	4 (15%)
	Artifact precluding evaluation^e^	3 (3%)	0 (0%)

^*a*^When comparing the susceptibility weighted imaging (SWI) scans, it is highly likely that the microbleed pathophysiologically changed from the first to the second scan

^b^The microbleed looks different at *t1* and *t2*, but uncertainty remains whether the different appearance reflects a pathophysiological or a technical difference

^*c*^Both possible and definite microbleed can be advocated

^d^Visible at both scans, but classified differently due to an artifact. For example, microbleed’s appearance changes from definite to possible due to blurring by an artifact

^e^Artifact making the microbleed invisible at one scan while visible at the other

To maximize the quality of the classification, we reviewed each microbleed with a possible MRI technical or pathophysiological difference (i.e., those classified as (possible) pathophysiological or artificial *cause of difference*) with an MR physicist (MM, 11 years of experience), incorporating additional MR sequences, if required (FLAIR, T1, T2, DWI).

### Blinding

In order to avoid observer bias due to influence of clinical information on the classification of candidate microbleeds, the neuroradiologists and MR physicist interpreting the SWI scans (AE, BG, MM) had no access to clinical information.

### Statistical analyses

Data were analyzed using SPSS 25 and STATA 15 statistical software. We performed Kolmogorov–Smirnov test to test normality of the distribution of the number of microbleeds per patient. As they were not normally distributed, we performed Wilcoxon signed-rank tests to compare numbers of microbleeds between the time-points. Within the subset of *single-scan* possible microbleeds at *t1*, we aimed to identify the most relevant predictors of the classification at *t2*. To identify predictors of the classification at *t2*, we performed two-level multinomial logistic regression with random effects for patient ID. Patient-level variables were age (years), gender, GCS at arrival at the emergency department, signs of elevated intracranial pressure at the emergency department (yes/no), normalized extra-axial injury score, number of intraparenchymal hemorrhages, number of non-hemorrhagic contusions, and total number of definite microbleeds at *t1* (*single-scan*). Microbleed level variables were shape (elongated versus spherical), distance from the inner surface of the skull (mm), and number of definite microbleeds at *t1* (*single-scan*) within 2 cm of the microbleed under evaluation. The dependent variable in these analyses was the classification at *t2*, using staying possible as the reference category. The variables with *p* < 0.2 in the univariable analyses were included in multivariable analysis. Spearman *r* between the independent variables was < 0.3 for each pair of variables. Microbleeds in the MARS region’s deep and periventricular white matter, basal ganglia, thalamus, and internal and external capsule were excluded from the analyses on extra-axial injury score, as their extra-axial injury score is zero by definition. Because of the nominal nature of the variable “MARS region,” we separately evaluated differences in the classification at *t2* between anatomical locations, using Kruskal–Wallis test with Bonferroni-corrected post hoc pairwise comparisons. Values are expressed as median (interquartile range) unless otherwise stated. Differences with *p* < 0.05 were considered significant.

## Results

A total of 1038 non-contiguous microbleeds were detected, 173 of which were possible microbleeds (Table 3).

### Longitudinal evolution of possible microbleeds

Ninety-three of the 173 (53.8%) possible microbleeds at *t1* corresponded to no microbleed at *t2*, 53 (30.6%) to a possible, and 27 (15.6%) to a definite microbleed.

### Predictors of the longitudinal evolution of possible microbleeds

In univariable but not in multivariable analyses, the odds of corresponding to no microbleed at *t2* were higher in males (Table 5).

**Table.5:** Predictors of the *t2*-classification of possible microbleeds at *t1*

	OR (95% CI, *p*) of classification as “no microbleed” at *t2*		OR (95% CI, *p*) of classification as “definite microbleed” at *t2*	
	Univariable analyses	Multivariable analysis	Univariable analyses	Multivariable analysis
Elongated shape^a^	.56 (.17–1.87, .350)	-	1.11 (.24–5.17, .892)	-
Distance from inner surface of skull (mm)	1.02 (.97–1.08, .368)	-	1.02 (.96–1.09, .527)	-
Number of definite microbleeds within 2 cm of the microbleed under evaluation^b^	.97 (.83–1.12, .638)	.95 (.80–1.13, .550)	1.23 (1.04–1.45, .015)*	1.22 (1.02–1.45, .028)*
Extra-axial injury score^c^	1.07 (.77–1.50, .680)	1.34 (.89–2.03, .159)	1.54 (1.03–2.29, .034)*	1.77 (1.09–2.85, .020)*
Number of intraparenchymal hemorrhages^c^	.99 (.42–2.35, .987)	-	.56 (.11–2.83, .484)	-
Number of non-hemorrhagic contusions^c^	.41 (.07–2.28, .307)	-	-^f^	-
Total number of definite microbleeds at *t1*^b^	1.00 (.99–1.02, .671)	-	1.01 (.98–1.03, .547)	-
GCS at arrival at the ED	.90 (.74–1.10, .300)	.90 (.74–1.10, .319)	1.20 (.98–1.47, .072)^(*)^	1.19 (.99–1.43, .071)
Male gender	2.90 (1.10–7.68, .032)*	3.08 (.98–9.68, .054)	1.43 (.35–5.81, .619)	1.22 (.33–4.46, .765)
Age (years)^d^	.99 (.96–1.02, .519)	-	.97 (.93–1.02, .221)	-
Signs of elevated intracranial pressure at the ED^e^	.84 (.45–1.58, .591)	-	.53 (no convergence achieved)	-

Results of two-level multinomial logistic regression analyses with random effects for patient ID, using the classification at *t2* as the dependent variable. Staying possible is the reference category. The multivariable analyses include all independent variables with *p* < .2 in the univariable analyses (see *Online Resource* for a list of the independent variables). For example, for each additional definite microbleed within 2 cm of the possible microbleed under evaluation, the odds of being classified as a definite microbleed at *t2* independently increased with a factor 1.22

^*^p < .05

^(*)^included in multivariable analysis, because *p* < .2 in univariable analysis

^a^Defined as longest axis ≥ 2* short axis

^b^At *single-scan* evaluation

^c^In the same MARS region at *t1*, normalized to the volume of the MARS region in MNI space

^d^At the day of trauma

^e^Generalized or hemispheric edema with effacement of sulci, compressed ventricles or basal cisterns and midline shift > 5 mm (yes/no)

^f^None of the possible microbleeds at *t1* in a MARS region with non-hemorragic contusion, corresponded to a definite microbleed at *t2*

*GCS* Glasgow Coma Scale score, *ED* emergency department

Both in univariable and in multivariable analyses, the odds of corresponding to a definite microbleed at *t2* were higher for microbleeds in a MARS-region with a higher extra-axial injury score, and for microbleeds with a larger number of definite microbleeds within 2 cm of the microbleed under evaluation (Table 5).

**Fig. 2:**
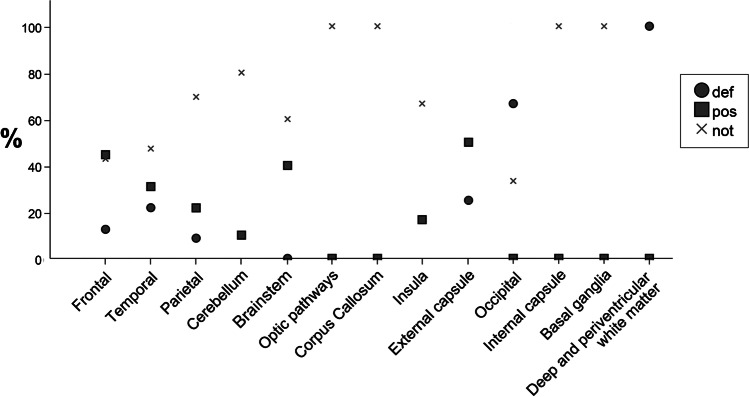
Anatomical distribution of possible microbleeds at *t1* corresponding to no, possible, and definite microbleeds at *t2*. The x-axis shows the anatomical regions in descending order of number of possible microbleeds at *t1*. Thalamus is not on the x-axis, as it contained no possible microbleeds at *t1*. The y-axis shows per region the percentage of possible microbleeds at *t1* that was classified as definite (circle), possible (square), or no (cross) microbleed at *t2*. *t1*: 3 (2–5) weeks after TBI, *t2*: 26 (25–28) weeks after TBI, *def*: definite microbleed, *pos*: possible microbleed, *not*: no microbleed

Figure 2 shows the anatomical distribution of possible microbleeds at *t1* corresponding to no, possible, and definite microbleeds at *t2*. Overall, the classification at *t2* differed between the regions (*p* < 0.05). No difference between individual pairs of anatomical regions was demonstrated.

### Causes of differences

**Fig. 3:**
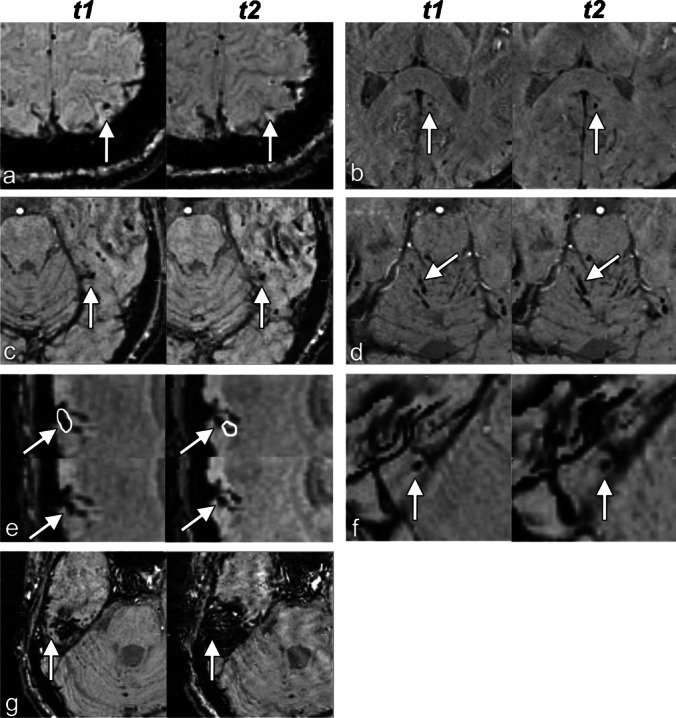
Examples of possible microbleeds at *t1* classified differently at *t2*. **a** Pathophysiological difference (disappearance); **b** possible pathophysiological difference, with slightly decreased signal intensity and increased blooming at *t2*, which may be caused by a technical difference between the scans or by a pathophysiological change; **c** equivocal classification, especially at *t1* this configuration can as well be classified as a possible microbleed as as a continuation of the blood vessel medial to it; **d** misinterpreted otherwise: this subarachnoid blood was mistaken for a possible microbleed at *t1*; **e** misregistration or missegmentation: at both time-points, this possible microbleed was segmented too small; the segmentations did not overlap, resulting in a false mismatch in the automatic comparison step; **f** artifact hampering the interpretation (susceptibility at air-tissue interface); **g** artifact precluding evaluation at *t2* (susceptibility at air-tissue interface). Images are axial images. Arrows point to the microbleeds discussed. In **e**, the automatic segmentation resulted in the closed curves. *t1*: 3 (2–5) weeks after TBI, *t2*: 26 (25–28) weeks after TBI

Table 4 summarizes the frequency of the various causes of classification differences. Figure 3 illustrates each etiological category.

#### From possible to no microbleed

The classification difference of 78 of the 93 possible microbleeds corresponding to no microbleed at *t2* was attributable to misinterpretation or to an artifact (84%, Table 4).

The artifacts hampering or precluding the interpretation were due to susceptibility at an air-tissue interface (16), pulsation (14), motion (8), and susceptibility induced by extra-axial blood (1). The misinterpretations reflected a vessel (26), subarachnoid blood (8), subarachnoid blood or a vessel (3), metal (1), and an unclassified artifact (1) that were erroneously taken for a possible microbleed. Five of the pathophysiologically changed microbleeds had decreased in diameter, while 2 had completely disappeared.

#### From possible to definite microbleed

The classification difference of 22 of the 27 possible microbleeds corresponding to a definite microbleed at *t2* was attributable to a pathophysiological or possible pathophysiological difference, to misregistration/missegmentation or to misinterpretation (81%, Table 4). All of the 5 pathophysiologically changed microbleeds had grown over time. The artifacts hampering the interpretation were due to pulsation (2), motion (1), and susceptibility at an air-tissue interface (1). The misinterpretations were a microbleed where a vessel (3) or subarachnoid blood (1) was erroneously considered to be in the differential diagnosis, and one misinterpretation could not be explained.

## Discussion

We have shown that 16% of the possible microbleeds at baseline correspond to a definite microbleed at follow-up, while 54% correspond to no microbleed, and uncertainty remains in 31%.

We evaluated possible microbleeds after TBI with 3-T SWI in a setting reflecting radiological practice, i.e., interpretation of possible microbleeds without any information on the findings at follow-up. Previous reports on possible microbleeds evaluate non-traumatic microbleeds assessed with GRE T2*-weighted 1.5-T MRI. Their objective differs from ours: they focus on the interobserver agreement at a single time-point, while we evaluate the temporal evolution of possible microbleeds [13, 14]. Whereas previous studies report patient-based numbers and include a total of 24–46 [14] and 52–63 [13] possible microbleeds, we report on 173 individual possible microbleeds, allowing evaluation of predictors of the longitudinal evolution of possible microbleeds.

### Predictors of the longitudinal evolution of possible microbleeds

The predictors of the longitudinal evolution of possible microbleeds comprise anatomical, demographic, and injury-related variables. The associations probably rely on a combination of interpretational and pathophysiological factors.

Only the minority of possible microbleeds evolve into a definite microbleed. This happens most frequently to possible microbleeds surrounded by many definite microbleeds. This may be due to an unfavorable micro-environment, caused by an inflammatory response to blood-derived substances in the perifocal zone, especially free iron and heme [23]. In this setting, a minimal amount of blood products, having oozed into the parenchyma at *t1*, barely visible on SWI and thus classified as a possible microbleed, could progress into a definite microbleed at *t2*. While in a favorable micro-environment, this minimal amount of blood products could have been resorbed. Also, a higher extra-axial injury load is independently predictive of turning into a definite microbleed. This may be related to a loss of compression over time, as extra-axial injury mostly decreases from baseline to follow-up. However, also the reliability of SWI interpretation may improve with a reduction of extra-axial injury over time, thus reducing the necessity of classifying a microbleed as possible. Alternatively, the etiology of a possible microbleed near many definite microbleeds or near extra-axial injury might differ from other microbleeds.

Half of the possible microbleeds correspond to no microbleed at follow-up. This may reflect resorption of minimal amounts of blood products in some cases, but may very well reflect the intrinsic uncertainty of the classification “possible” in the majority [24]. Though possible microbleeds in males have increased odds of disappearing, this relation is not causal, as in multivariable analyses this relation is not significant. In the optic pathways, internal capsule, basal ganglia, and corpus callosum, all of the possible microbleeds disappeared at *t2*. However, the numbers are too low to draw conclusions about the relation between anatomical location and the course of possible microbleeds.

As the sparsity of statistical differences may have been caused by the limited number of possible microbleeds per region and per candidate predictor, we also discuss possible inferences from trends. First, we discuss trends appearing from the regression analyses (Table 5), and then we zoom in to the anatomical regions (Fig. 2 and Table 3).

Whereas a higher extra-axial injury load is independently predictive of turning into a definite microbleed, there is also a trend of a higher extra-axial injury load being predictive of turning into no microbleed (Table 5). This paradox indicates that in regions with more extra-axial injury, fewer possible microbleeds stay possible. This suggests that the role of extra-axial injury may not be a pathophysiological influence on microbleeds, but rather an influence on the reliability of microbleed evaluation. Indeed, the reliability of SWI interpretation may improve with the resorption of extra-axial injury over time, thus reducing the necessity of classifying a microbleed as possible: the criteria for classifying it as a definite microbleed or as no microbleed are more easily met in the absence of interfering concomitant extra-axial lesions.

We limit our discussion of the location of possible microbleeds to the regions with > 5 possible microbleeds, i.e., frontal, temporal, parietal, and cerebellum. Mainly due to their different locations in relation to the skull and the dural folds, the predominant mechanisms of injury differ between these regions, with the frontal and temporal lobe being more similar than the parietal lobe and cerebellum. Also the course of possible microbleeds is similar in the frontal and temporal lobes, and different in the parietal lobe and cerebellum (Fig. 2). On the one hand, this may suggest pathophysiological differences between microbleeds at the different locations. On the other hand, possible pathophysiological differences of the microbleeds themselves cannot be unraveled from the role of concomitant injury, which is unevenly distributed over the regions (Table 3). The cerebellum and parietal region are the regions with the largest proportion of microbleeds being classified as possible. In line with the discussion above, these same regions are the regions with the highest subarachnoid hemorrhage load (Table 3), which may have caused the proportion of microbleeds being classified as possible in these regions to be high. Indeed, in these two regions, the vast majority of possible microbleeds turned into no microbleed at follow-up, whereas in the regions with less subarachnoid hemorrhage, i.e., the temporal and to a lesser extent the frontal region, a substantial proportion of the possible microbleeds turned into a definite microbleed at follow-up (Fig. 2). This suggests that the presence of concomitant subarachnoid hemorrhage renders the interpretation of microbleeds more uncertain, but that the uncertainty fades when the subarachnoid blood is resorbed. Intraparenchymous hemorrhage on the other hand may be associated with remaining uncertainty on the classification of microbleeds, even after evolution of the intraparenchymous hematoma: the vast majority of intraparenchymous hemorrhages was observed in the frontal and temporal lobe (Table 3), where a substantial proportion of possible microbleeds stayed possible at follow-up (Fig. 2). In conclusion, a follow-up SWI scan could be advocated especially for possible microbleeds located in regions with subarachnoid hemorrhage, and the yield of a follow-up scan may be lower for possible microbleeds located in regions with intraparenchymous hemorrhage.

### Recommendations for radiological practice

In radiological practice, possible microbleeds are encountered on a daily basis. Though for the majority of patients the interpretation of possible microbleeds will not change management, prognostication, or trauma grading, for some patients it will. The accuracy of the interpretation of possible microbleeds is especially important, if they affect the grading of traumatic/diffuse axonal injury, or if they are in patients with few or no definite microbleeds, especially in forensic cases, or in patients with persistent post-traumatic symptoms to which the contribution of traumatic and non-traumatic factors need to be disentangled.

This is a first step in the answer to the question of how to deal with a possible microbleed on a single SWI scan in a clinical radiological setting. On the one hand, only 16% of the possible microbleeds at *t1* turns into a definite microbleed, and therefore possible microbleeds may be regarded as no microbleed. On the other hand, uncertainty remains in 31%: on two separate scans they are classified as a possible microbleed. As adding a second scan did result into a more certain classification (definite or no microbleed) of 69% of the possible microbleeds on a single scan, a second SWI scan could be advocated in case of clinical consequences. However, even though classified by two neuroradiologists, as many as 25% of the possible microbleeds were misinterpreted at single scan evaluation. In addition to the intrinsic difficulty of the evaluation of possible microbleeds, this may also be due to the large total amount of microbleeds and false-positive CAD detections to be interpreted (we misinterpreted 4% of all of the possible and definite microbleeds, and circa 1.8% of all of the locations that were detected by the CAD system (denominator inferred from Van den Heuvel et al. [9])). Therefore, before performing a follow-up scan, possible microbleeds merit a second inspection to make sure that they cannot be reclassified as a definite or no microbleed: the cause of classification difference between the timepoints is misinterpretation in as many as 25% of the possible microbleeds. Alternatively, the decision on an individual possible microbleed could incline toward a microbleed if it is near many other microbleeds or in a region with extra-axial injury.

### Limitations

Of the screened patients, only a small proportion was finally included in the study. This was mainly due to lack of informed consent or decease before the first MRI, reflecting the difficulty of patient inclusion from the ICU. Of the remaining patients, 8 had to be excluded due to motion artifacts, a well-known challenge of performing MRI in patients with severe TBI. Both effects may have introduced a selection bias toward a lower injury severity, possibly with lower microbleed loads.

A single neuroradiologist evaluated a substantial proportion of the microbleeds. Aware of the resulting vulnerability to inter-rater variability-based bias, the second neuroradiologist was consulted in case of the slightest doubt. Additionally, the second neuroradiologist reviewed a random sample of 30 of the other microbleeds and agreed on all of them. The automatic comparison between the time-points could be viewed as a surrogate co-reader, prompting to re-evaluate each differing microbleed in consensus meetings.

Another limitation is that at the *single-scan* stage, we only evaluated SWI. In the *post-comparison* stage, we used additional sequences. We had no access to the SWI-phase images^7^.

The lack of histopathologic correlation limits the interpretation of the longitudinal findings. Ten percent of the possible microbleeds showed evidence of a pathophysiological change over time, but they were too few to draw conclusions on predictors of their evolution. As no repeat SWI scan at *t1* was available, the effects of technique (re-test reliability) cannot be separated from the physical longitudinal evolution in the search for predictors of the classification at follow-up. This is a purely diagnostic study. The number of patients is insufficient to perform meaningful patient-wise analyses and to evaluate the clinical significance of our findings. Though the data acquisition on a single scanner improved the data homogeneity, it limits the generalizability of our findings to clinical practice.

### Conclusion

Sixty-nine percent of the possible microbleeds at baseline are classified differently at follow-up. Only 16% of them represent a definite microbleed at follow-up.

The longitudinal evolution differs between locations and micro-environments. Closeness to extra-axial injury and closeness to definite microbleeds are independently predictive of corresponding to a definite microbleed at follow-up. The reclassification level differs between anatomical locations, though no difference between individual pairs of anatomical regions was demonstrated. Ten percent of the classification differences between baseline and follow-up reflect a pathophysiological change.

Future research is needed to evaluate the pathophysiological and interpretational backgrounds of our findings.

Our results support disregarding possible microbleeds in the absence of clinical consequences. In case of clinical consequences, however, a follow-up SWI scan could be obtained to exclude evolution into a definite microbleed, especially if the possible microbleed is surrounded by many definite microbleeds, or is in a region with extra-axial injury.

## Supplementary Information

Below is the link to the electronic supplementary material.

Supplementary file1 (DOCX 29.2 KB)

## Data Availability

Upon request.

## Code availability

Upon request.
