# Supplementary material for: The radiological interpretation of possible microbleeds after moderate or severe traumatic brain injury: a longitudinal study
Source: Neuroradiology. 2021 Oct 31;64(6):1145–56. doi: 10.1007/s00234-021-02839-z (PMC9117345; doi:10.1007/s00234-021-02839-z)
Supplement: Supplementary file 1 — Supplementary file1 (DOCX 29.2 KB) [file 234_2021_2839_MOESM1_ESM.docx]

**Online Resource**

*Supplement to Van der Eerden AW, Van den Heuvel TLA, Maas MC, et al (2021)* ***The Radiological Interpretation of Possible Microbleeds after Moderate or Severe Traumatic Brain Injury****. Neuroradiology.* [*https://doi.org/10.1007/s00234-021-02839-z*](https://doi.org/10.1007/s00234-021-02839-z)

In the following pages we describe details on the scoring procedures of concomitant injury and of microbleeds.

**Index of Supplementary Information**

- Scoring Procedure of Concomitant Injury
- Additional Details on Microbleed Evaluation
- References of Supplementary Information

**Scoring Procedure of Concomitant Injury**

We used a clinical PACS viewing system to record any concomitant injury, i.e. intracranial traumatic injury other than microbleeds, using all available MR-sequences at *t1*. We counted the number of non-hemorrhagic contusions and the number of intraparenchymal hemorrhages (including hemorrhagic contusions) in each Microbleed Anatomical Rating Scale-based region (MARS-region), ignoring any satellite lesions. We also calculated an extra-axial injury score based on the presence and amount of subarachnoid, subdural and extradural hemorrhage in contact with each MARS-region. Each MARS-region obtained a maximum of 3 points: 1 point for contact with an epidural hematoma of ≥1 cm thickness [1], using ≥2 cm as a threshold for epidural hematomas at the temporal pole if they were juxtaposed to the sphenoparietal sinus, limited laterally by the sphenotemporal suture and medially by the orbital fissure, and not extending above the lesser sphenoid wing, as these are usually treated conservatively because they tend to have a benign natural history [2]. 1 point for contact with a subdural hematoma of ≥1 cm thickness [3]. And a maximum of 1 point for contact with subarachnoid hemorrhage: 1/3 point for modified Fischer grade 2, 2/3 point for grade 3 and 1 point for grade 4 [4,5]. As modified Fischer grade 4 implies diffuse injury, in a patient with grade 4 subarachnoid hemorrhage each MARS-region obtained 1 point for subarachnoid hemorrhage. The numbers of contusions and intraparenchymal hemorrhages, as well as the extra-axial injury scores were normalized to the volume of the MARS-region they were in by dividing them by the volume of the MARS-region in Montreal Neurological Institute (MNI) space [6,7].

**Additional Details on Microbleed Evaluation**

Bilateral symmetric lesions in the basal ganglia were not recorded as microbleeds since these are most likely calcifications [8].

We recorded the subclassification ‘elongated microbleed’ in case of microbleeds with long axis ≥2*short axis, otherwise matching all of the criteria of a CMB.

A random sample of thirty of the microbleeds and the false-positive CAD-detections, the classification of which X1 in the *single-scan* evaluation step assumed to be unequivocal, was reviewed by X2. She agreed on all of them. This suggests that the assumption of unequivocality of the classification of this subset of microbleeds was justified.

Each microbleed was automatically segmented using intensity-based volume-constrained region growing. Errors made in the automatic segmentation step were manually corrected by X1. Each microbleed was allocated to a MARS-region based on automatic registration of the MARS atlas to the segmentations, using the non-linear registration tool FNIRT [9,10]. Microbleeds located at a border were distributed over the involved MARS-regions in proportion to the segmentation’s volume in the MARS-regions. Microbleeds allocated to MARS-regions vulnerable to misregistration, such as occipital and cerebellar, were reviewed for need of manual re-allocation. If these were re-allocated to more than one region, they were counted as evenly distributed, e.g. a microbleed at the occipito-temporal border was counted as .5 occipital microbleed and .5 temporal microbleed.

**References of Supplementary Information**

1. De Souza M, Moncure M, Lansford T, et al (2007) Nonoperative management of epidural hematomas and subdural hematomas: is it safe in lesions measuring one centimeter or less? J Trauma 63(2):370-372

2. [Gean](https://pubmed.ncbi.nlm.nih.gov/?term=Gean+AD&cauthor_id=20713606) AD, [Fischbein](https://pubmed.ncbi.nlm.nih.gov/?term=Fischbein+NJ&cauthor_id=20713606) NJ, [Purcell](https://pubmed.ncbi.nlm.nih.gov/?term=Purcell+DD&cauthor_id=20713606) DD, [Aiken](https://pubmed.ncbi.nlm.nih.gov/?term=Aiken+AH&cauthor_id=20713606) AH, [Manley](https://pubmed.ncbi.nlm.nih.gov/?term=Manley+GT&cauthor_id=20713606) GT, [Stiver](https://pubmed.ncbi.nlm.nih.gov/?term=Stiver+SI&cauthor_id=20713606) SI (2010) Benign anterior temporal epidural hematoma: indolent lesion with a characteristic CT imaging appearance after blunt head trauma. Radiology 257(1):212-218

3. [Evans](https://pubmed.ncbi.nlm.nih.gov/?term=Evans+JA&cauthor_id=25109659) JA, [Bailey](https://pubmed.ncbi.nlm.nih.gov/?term=Bailey+M&cauthor_id=25109659) M, [Vail](https://pubmed.ncbi.nlm.nih.gov/?term=Vail+A&cauthor_id=25109659) A, [Tyrrell](https://pubmed.ncbi.nlm.nih.gov/?term=Tyrrell+PJ&cauthor_id=25109659) PJ, [Parry-Jones](https://pubmed.ncbi.nlm.nih.gov/?term=Parry-Jones+AR&cauthor_id=25109659) AR, [Patel](https://pubmed.ncbi.nlm.nih.gov/?term=Patel+HC&cauthor_id=25109659) HC (2015) A simple tool to identify elderly patients with a surgically important acute subdural haematoma. Injury 46(1):76-79

4. [Steyerberg EW](https://www.ncbi.nlm.nih.gov/pubmed/?term=Steyerberg%20EW%5BAuthor%5D&cauthor=true&cauthor_uid=18684008), [Mushkudiani N](https://www.ncbi.nlm.nih.gov/pubmed/?term=Mushkudiani%20N%5BAuthor%5D&cauthor=true&cauthor_uid=18684008), [Perel P](https://www.ncbi.nlm.nih.gov/pubmed/?term=Perel%20P%5BAuthor%5D&cauthor=true&cauthor_uid=18684008), et al (2008) Predicting outcome after traumatic brain injury: development and international validation of prognostic scores based on admission characteristics. PLoS Med 5(8):e165

5. [Chieregato A](file:///C:\pubmed\%3fterm=Chieregato%20A%5bAuthor%5d&cauthor=true&cauthor_uid=15792505), [Fainardi E](file:///C:\pubmed\%3fterm=Fainardi%20E%5bAuthor%5d&cauthor=true&cauthor_uid=15792505), [Morselli-Labate AM](file:///C:\pubmed\%3fterm=Morselli-Labate%20AM%5bAuthor%5d&cauthor=true&cauthor_uid=15792505), et al (2005) Factors associated with neurological outcome and lesion progression in traumatic subarachnoid hemorrhage patients. Neurosurgery 56(4):671-680

6. MNI ICBM 152 non-linear 6th generation symmetric average brain stereotaxic registration model. <http://nist.mni.mcgill.ca/?p=858> Accessed December, 2017

7. Grabner G, Janke AL, Budge MM, Smith D, Pruessner J, Collins DL (2006) Symmetric atlasing and model based segmentation: an application to the hippocampus in older adults. In: Larsen R, Nielsen M, Sporring J (eds) Medical Image Computing and Computer-Assisted Intervention – MICCAI 2006. Lecture Notes in Computer Science, vol 4191. Springer, Berlin Heidelberg New York

8. Tsushima Y, Endo K (2006) Hypointensities in the brain on T2*-weighted gradient-echo magnetic resonance imaging. Curr Probl Diagn Radiol 4:140–150

9. FSL FNIRT. <https://fsl.fmrib.ox.ac.uk/fsl/fslwiki/FNIRT>. Accessed December 2017

10. Jenkinson M, Beckmann CF, Behrens TE, Woolrich MW, Smith SM (2012) FSL. Neuroimage 62(2):782-790
